# The improvement strategy of fresh produce supply chain resilience based on extenics

**DOI:** 10.1371/journal.pone.0309008

**Published:** 2024-09-30

**Authors:** Zhangzheyi Liao, Chaoling Li, Lin Lu, Xiaochun Luo

**Affiliations:** 1 School of Economics and Management, Guangxi Normal University, Guilin, China; 2 School of Economics and Management, Heilongjiang Bayi Agricultural University, Daqing, China; 3 School of Economics and Management, Nanjing University of Aeronautics and Astronautics, Nanjing, China; University of Agriculture Faisalabad, PAKISTAN

## Abstract

Nowadays, the world is in turmoil, climate and environmental problems are prominent, the import and export of fresh agricultural products are restricted, etc. The impact of the growing demand for fresh agricultural products and healthy lifestyle choices, and fresh agricultural products are essential for people’s daily life, which are perishable, fragile, seasonal, and other unstable factors. Therefore, when the fresh produce supply chain faces various pressures and difficulties, how to enhance the resilience of the supply chain against various problems and risks with flexible and multi-dimensional strategies and methods has become the focus of extensive attention. This kind of problem is a typical contradictory problem, and previous studies have failed to achieve good results. In this paper, based on extenics, we are able to one-dimensionalise the multi-dimensional contradictory problems and multi-dimensionalise the one-dimensional contradictory problems to solve such problems in a scientific and effective way. Firstly, taking fresh agricultural products supply chain enterprise M as the research object, we constructed the fresh agricultural products supply chain enterprise toughness system and identified the toughness state of each index. Secondly, we found the low-evaluation toughness indexes that need to be solved and constructed a extension model of incompatible problems of enterprise toughness. Thirdly, we analysed the objectives and conditions of toughness incompatible problems of fresh agricultural products supply chain enterprises numerically and quantitatively, and then, with the objective of toughness improvement, we analyzed the correlation of the condition basic-elements of incompatible problems and carried out extension transformations. Again, the objectives and conditions of the incompatible problems of fresh produce supply chain enterprises are analysed numerically and quantitatively, and with toughness enhancement as the objective, the correlation analysis and extension transformation of the condition basic-elements of the incompatible problems are implemented to generate the set of toughness enhancement strategies that can solve the incompatible problems in a multidimensional and scientific way. Finally, the optimal toughness enhancement strategies are selected through the superiority evaluation and composed into a new strategy to enhance the toughness of the fresh produce supply chain. Combined with extenics calculations and screening, a new strategy for supply chain resilience enhancement of fresh agricultural products was finally formed. The existing problems are solved from six aspects: product supply type, product demand, product supply efficiency, human resource quantity, production and processing equipment, and logistics guarantee ability. It provides a certain reference significance for the fresh agricultural products supply chain toughness enhancement, and helps enterprises to strengthen their competitiveness and sustainability through the enhancement of toughness.

## 1. Introduction

In today’s globalised and information-based business environment, there are many unstable factors, such as inter-regional conflicts and trade wars, public health emergencies and environmental and ecological pollution and other irresistible problems occur from time to time. Agriculture is the basic industry of national economy, and its stable and healthy development is of great significance [1]. In a complex world of interdependence and mutual influence, it is difficult to quickly restore the supply chain system to a normal state if any part of the system has problems. This will not only lead to large-scale disruption in the fresh agricultural products logistics supply chain, but may even paralyse the entire supply chain system, bringing huge economic losses to the participants of the entire supply chain. It can be seen that the resilience of fresh agricultural products supply chain has not only become one of the indispensable key factors for the normal production of people as well as the success of enterprises or the realisation of national strategic deployment. Due to the special characteristics of fresh agricultural products, such as easy to wear and tear, regionality, and strong product variability, fresh agricultural products have been deteriorating over time in terms of nutritional value, weight, flavour, and colour [2]. This makes its supply chain system have higher integrity and complexity compared with the supply chain system of other commodities, and the resistance and adaptability to exogenous shocks is weaker. In order to cope with this challenge, it is of great practical significance to conduct resilience evaluation research on fresh produce supply chain. Resilience evaluation refers to the comprehensive assessment of the structure, function and performance of the supply chain system, analysing its adaptability and stability under the changes of the external environment, so as to provide a scientific basis for supply chain management.

For the fresh agricultural products supply chain resilience enhancement strategy can be evaluated and explored from the following three main aspects. First, the resilience of the supply chain structure, fresh agricultural products supply chain structure including production, processing, storage, transport, sales and other links [3], need to ensure that in the face of market fluctuations, policy adjustments, natural disasters and other uncertainties, the links can be flexibly adapted to ensure the stable operation of the overall supply chain. Secondly, the resilience of supply chain function, fresh agricultural products supply chain function mainly includes information transmission [4], resource allocation [5], risk management [6] etc., which needs to have the ability to respond and adjust quickly when facing various problems to ensure the efficient operation of the supply chain. Finally, the resilience aspect of supply chain performance, fresh agricultural products supply chain performance mainly includes on-time delivery rate, inventory turnover rate, cost control [7–9] etc., which needs to have the ability to maintain good performance when facing pressure to ensure the overall competitiveness of the supply chain.

By understanding the various aspects of fresh produce supply chain resilience, we can better understand the strengths and weaknesses of the fresh produce supply chain system, so as to formulate targeted improvement measures to improve the supply chain’s ability to resist risk and cope with uncertainty. At the same time, the resilience enhancement strategy can also provide us with a decision-making basis to help managers and enterprises make more reasonable and effective strategic choices when facing market changes. Based on the principle of topology, the article seeks to find the most enhancement strategies for the resilience enhancement strategy of fresh agricultural products supply chain through the transformation of primitive logic.

In conclusion, the research on the resilience enhancement strategy of fresh produce supply chain is of great practical significance. By improving the resilience of fresh agricultural products supply chain, it can strengthen the scale, standardisation and intensification of fresh agricultural products production, break the information barriers between urban and rural areas and logistics barriers, make the fresh agricultural products supply chain more precise and standardised, better promote the effective docking and cooperation between the supply and demand of fresh agricultural products, and promote the sustainable development of agriculture and fresh agricultural products economy. This paper takes fresh produce supply chain enterprise M as an example, evaluates its supply chain resilience system and constructs and optimises the problem model for the resilience indicators that do not meet the standard. In summary, the article identifies the following two research objectives

(1) To construct the index system of fresh produce supply chain and clarify the relevant calculation methods, so as to provide support for effectively selecting the optimisation index and the most optimal fresh produce supply chain;

(2) Combined with the resilience evaluation indexes, the intelligent selection optimisation method of fresh produce supply chain is proposed through data mining and computation, which is free from the inherent stereotypes of strategy generation and realises the strategy generation of resilience optimisation of fresh produce supply chain.

This study makes three major contributions to the field of fresh produce supply chain: There are three major contributions to the field of supply chain research. We address a question that has been neglected and understudied in previous research: how to improve fresh produce supply chain resilience? Firstly, based on the past fresh agricultural products supply chain resilience system, this paper carries out scientific rationality innovation and construction, enriches the relevant theories and methods of fresh agricultural products supply chain research, determines the feasibility of the system, and provides new practical guidance and valuable insights for fresh agricultural products supply chain resilience research. Secondly, based on the deep reinforcement learning algorithm, this paper proposes a specific algorithm for optimising the fresh agricultural products supply chain. Finally, this paper optimises and restructures the supply chain toughness of fresh agricultural products based on the characteristics of fresh agricultural products supply chain based on the knowledge of extenics, and the innovation of toughness enhancement strategy improves the objectivity and effectiveness of the supply chain.

## 2. Literature review

### 2.1. Fresh produce supply chain research status

The supply chain is closely linked to a wide range of participants, such as producers, processors, wholesalers, retailers, consumers, governments and non-profit organisations. It covers multiple steps in the planting, breeding, harvesting, processing, storage, transport and marketing of fresh produce. Research on fresh produce supply chains generally began in the 1990s, with scholars focusing on exploring the challenges faced by agricultural logistics and marketing models, and actively seeking solutions and innovative approaches to promote their development. However, there is a relative lack of research on the resilience of fresh produce supply chains. Mari [10] argued that significant post-harvest losses occur in the fresh produce supply chain. Perishability is one of the main factors affecting the quality of fruits and vegetables. Xiao [11] considered the problem of perishability in the fresh produce supply chain that results in some products being unsaleable, analysed two business models: the pull model and the push model, and investigated the optimal decisions of the supply chain members. Porat [12] in order to cope with the problem of losses during the retailing and consumption period, made a study of the logistic and cold chain management, retailing packaging and technological innovations, encouraging post-harvest researchers to be more actively involved in logistics and food supply chain operations and conducting multidisciplinary research. Hughes [13] defined fresh produce supply chain in the direction of supply chain synergy and co-operation. The fresh produce supply chain is a network of business generated from its production to distribution process, that is, a collection of transactions occurring in the process of from farmers to clients. By combining the fresh produce The collection of resources in the supply chain forms a kind of chain network with transaction function, and the individuals in the chain can reach the partnership. In order to achieve an efficient fresh produce supply chain, the freshness and loss of fresh produce are guaranteed within a certain controllable range. Cai [14] pointed out that in order to guarantee the quality and quantity of the produce, the distributors need to consider the order, freshness work, and selling price, as well as the producer’s wholesale price, the cost of freshness, and the damage of transport. In addition, the producer needs to determine the wholesale price based on the distributor’s order quantity. Kamble [15] argued that with the growing importance of agricultural supply chains, agricultural supply chains are designed and operated more tightly regulated and closely monitored to adapt to new regulatory environments and consumer demands, and that further research and development of new models and methodologies are needed to meet the complexity and diversity of agricultural supply chains. Accorsi [16] considered the food supply chain as an ecosystem, and using a regional potato supply chain as an example, the study found interdependencies between infrastructure, production, distribution, and environmental resources. Besik [17] developed a model of an integrated, multilevel competitive agricultural supply chain network in which agribusinesses and processors compete to sell their differentiated products. Carstens [18] summarised the pathways through which fresh produce can be contaminated in the supply chain, both pre-harvest and post-harvest. Yu [19] investigated the impact of outsourcing patterns on supply chain decision-making and profitability by modelling the game of fresh produce supply chains, driven by the widespread use of outsourcing of cold-chain services. Omar [20] reviewed the crop-based agricultural production and distribution planning field and explored the main contributions in the field, exploring the applicable models for agricultural supply chains in different environments from the perspective of fresh and non-fresh agricultural products.

### 2.2. State of the art in supply chain resilience research

In the 2000s, the study of resilient supply chains gradually attracted extensive attention from scholars. With the continuous development and change of the global economy, the importance of supply chain management has become more and more prominent. Resilient supply chain as an effective means of coping with uncertainty and risk has received a great deal of attention from academics. Wieland [21] argues that resilience is not only related to the ability of the system to "bounce back" after an impeding event, but also the ability to adapt and transform, and that resilience of the supply chain is no longer understood as stability but as adaptation and transformation. Hosseini [22] introduced the concept of resilience, which refers to the ability to be resistant enough to withstand disruptions and recover quickly from them. Taghikhah [23] implemented an extension of the new concept of supply chains for agricultural products and modelled the adaptive behaviours of farmers, food processors, retailers, and customers. Gerken [24] synthesised how pest management ("IPM") strategies may help to improve the management of pests. IPM) strategies may help to improve the adaptive capacity and resilience of managing agricultural supply chains under climate change. Aslam [25] analysed the role of supply chain flexibility in the development of supply chain resilience. Aboah [26] identified flexibility, collaboration, adaptability and resourcefulness as key elements in assessing resilience at the level of the individual chain actors, and adaptability as a general level resilience assessment of the food system as a relevant element as it takes into account changes in the state of supply chain resilience Leat [27] explored the importance of the stability and sustainability of the pork supply chain, as one of the key industries in the region, for ensuring food security and promoting economic development. Anastasiadis [28] viewed the Greek wine supply chain as a systematic and holistic approach from the grapes to the shelf process; the product and the complex flow of information; wine supply chain and stakeholders and concluded that improving the resilience of the Greek wine industry remains an important issue. Feng [29] analysed the risk factors of the fresh grape supply chain from the perspective of sustainable development and assessed the risk level using an optimised BP neural network to provide recommendations for constructing a fresh grape supply chain with controllable risk and sustainable development. Marusak [30] explores how regionalised food supply chains can increase the resilience of the US food supply system, using logistics best practices to provide efficient and reliable distribution to consumers under normal conditions and during disasters in response to large-scale public problems like the COVID-19 pandemic. Boyaci- Gunduz [31] discusses panic buying observed during the crisis, food shortages and price spikes, and a review of food security and sustainability emphasises the importance of supply chain resilience. However, during development, we face a number of key risks and challenges such as climate impacts, fluctuating market demand, and transport depletion. To cope with these risks, we need to build a resilient fresh produce supply chain to ensure stable operations in the face of uncertainty. Wieland [32] used SEM to empirically investigate that communication and collaborative relationships have a positive impact on resilience, whereas integration does not have a significant effect. Scholten [33] found that information sharing, collaborative communication, co-created knowledge, and joint relational endeavours increase supply chain resilience by increasing visibility, speed, and flexibility. Ambulkar [34] found that firms with supply chain disruptions need to have the ability to reconfigure their resources or have a risk management resource infrastructure to foster resilience. Brandon-Jones [35] found that supply chain connectivity and information sharing resources lead to supply chain visibility capabilities to increase resilience.

### 2.3. Current status of research on extenics

Extenics [36, 37] is a wide-ranging and original transversal discipline that encompasses mathematics, philosophy, and engineering. It provides some new possibilities as well as methods and ideas for solving specific paradoxical problems in a more multidimensional and high-dimensional perspective with formal models. Cai, Yang [38] and others summarised the Extenics in theory into three major categories: Basic-element theory, Extension set theory and Extension Logic. Based on the primitive expansion model, Li [39] argued that the characteristics of things are collected through information technology, and then systematically expanded many innovative thinking directions. Zhou [40] helped college students to adapt to their lives and conduct research by analysing Matter-element, Affair-element and Relation-element of the Basic-element theory. Ruo [41] applied the Basic-element theory and investigated intelligent knowledge representation based on this theory method, which studies the characteristics of things and their corresponding characteristic quantities as a whole, uses primitive elements to formally describe things, behaviours and relationships, and builds an extended model to represent the knowledge. Li [42] in order to solve the problems in the traditional social network analysis model, uses the theory of Basic-element to use multidimensional object elements to represent the characteristics and nodes of a complex social network, and then later on integrates them in an advantageous way.

### 2.4. Research gap

In the current research, most of the research on the resilience elements of the fresh produce supply chain is to analyse and solve the problem of the supply resilience of individual varieties of fresh produce, or to carry out a simple quantitative analysis of a few resilience indicators of specific enterprises, which are not sufficiently influential and applicable to the resilience enhancement of other companies, and the selection of resilience indicators and resilience enhancement strategies lack a certain degree of scientific and comprehensive methodology only through a single research methodology as an entry point. Only through a single research method as an entry point, the selection of resilience indicators and resilience enhancement strategies lack a certain degree of science and comprehensiveness, failing to effectively help companies from a professional perspective to solve the contradictions between the current goals and the resilience conditions of the supply chain in all aspects.

Accordingly, in the current agricultural environment, it is particularly important to find ways to explore and resolve the incompatibility of supply chain resilience in the production of agricultural products. With the development of globalisation and the diversification of consumer demands, fresh produce supply chains are facing more and more challenges, which put forward higher requirements for supply chain resilience, i.e., the supply chain is able to maintain its operation and efficiency in the face of pressures and shocks so as to ensure a stable supply of fresh produce. In order to construct an enterprise resilience enhancement strategy, the article constructs a resilience enhancement strategy generation method with both expertise and applicability, which not only starts from the perspective of professional knowledge to understand the operation mechanism of the supply chain and the challenges it faces, but also takes into account the applicability of the enhancement strategy, which is able to adapt to different supply chain environments and enterprise needs. It provides customised resilience enhancement strategies for enterprises to help them cope with various challenges and improve the resilience of the supply chain, and provides scientific and effective guidelines for resilience enhancement strategies for fresh produce supply chain enterprises.

## 3. Model establishment and solution

### 3.1. Basic-element model

Assuming that the definition of the thing is M, the thing feature is C, the range of quantitative value of C is V. In this object meta-group, then it is the fresh produce supply chain resilience, and there are n resilience evaluation indexes for the fresh produce supply chain resilience of the feature *c*_*m*1_, *c*_*m*2_, ···, *c*_*mn*_, *O*_*m*_ about the quantitative values of *c*_*mi*_(*i* = 1,2,…*n*) the Matter-element matrix composed of *v*_*mi*_(*i* = 1,2,…*n*):

$$M=\left[\begin{array}{ccc} O_{m}, & c_{m1}, & v_{m1}\\ \; & c_{m2}, & v_{m2}\\ \; & \vdots & \vdots \\ \; & c_{mn}, & v_{mn} \end{array}\right]=\left(\begin{array}{ccc} O_{m}, & C_{m}, & V_{m} \end{array}\right).$$

### 3.2. Extension modelling of contradictory problems

For some relatively complex and difficult to use a basic-element clear description of things, extenics can be matter-element, affair-element and Relation-element combination into a composite element, the use of different descriptive characteristics of the basic-element more clearly to describe the establishment of understandable model.

In order to solve a contradictory problem, first of all, it is necessary to clearly define the targets *G* and conditions *L* to be achieved in order to solve the problem., the contradictory problem model is established:

P=G*L

When the objective *G* cannot be achieved under condition *L*, the problem is incompatible and is modelled as:

P=G↑L

the target *G* can be composed of multiple targets *g*_*i*_, i = (1, 2,…, m), the condition *L* can also be composed of multiple conditions *l*_*k*_, k = (1, 2,…, n), the core problem modelled as:

$$P=\left(g_{1}\wedge g_{2}\wedge \text{···}\wedge g_{m}\right)*\left(l_{1}\wedge l_{2}\wedge \text{···}\wedge l_{n}\right)$$

Take *X*_0_, (*X*_0_ ∈ *X*) as the positive domain and establish the compatibility function *k*(*x*) of the condition *l* with respect to *c*_0_. Denote *k*_0_(*p*) = *k*[*c*_0*t*_(*Z*_0_)] as the compatibility degree of the Incompatible problems *p*, and call the problem *p* a compatible problem when *k*_0_ (*p*) > 0; and call the problem *p* a critical problem if *k*_0_ (*p*) > 0. For the incompatibility problem *k*_0_ (*p*) < 0 of *p*, if there exists transformation *T* = (*T*_*U*_, *T*_*K*_, *T*_*L*_) such that $T_{K}K\left(T_{l0}l_{0}\right)=K\prime \left(T_{l0}l_{0}\right)=K^{\prime }\left(l_{0}^{\prime }\right)>0$ then *T* is called the solution transformation of the incompatible problem.

### 3.3. Integrated evaluation model

#### 3.3.1. Classical domains, section domains and objects to be evaluated

Classical domain: Divide the extension domain of fresh produce supply chain resilience index score, for the fresh produce supply chain resilience is divided into e levels (e = 1, 2,…, s), *O*_*oe*_ is the supply chain resilience evaluation level, *C*_*j*_ represents the evaluation of the resilience level of the indexes (j = l, 2,…,n), 〈*a*_*oej*_, *b*_*oej*_〉 denotes the interval of values of *V*_*oe*_ for *C*_*j*_ metric in the e-th resilience evaluation level. The classical domain *M*_*oe*_ has the following form:

$$M_{oe}=\left[\begin{array}{ccc} O_{oe,} & C_{j,} & V_{oej} \end{array}\right]=\left[\begin{array}{ccc} O_{oe} & c_{1} & {v_{oe}}_{1}\\ \; & c_{2} & v_{oe2}\\ \; & \vdots & \vdots \\ \; & c_{n} & v_{oen} \end{array}\right]=\left[\begin{array}{ccc} O_{oe} & c_{1} & \langle \begin{array}{cc} a_{oe1,} & b_{oe1} \end{array}\rangle \\ \; & c_{2} & \langle \begin{array}{cc} a_{oe2,} & b_{oe2} \end{array}\rangle \\ \; & \vdots & \vdots \\ \; & c_{n} & \langle \begin{array}{cc} a_{oen,} & b_{oen} \end{array}\rangle \end{array}\right]$$

Section domain: the value range of classical domains is merged to become the section domain, assuming that *O*_*P*_ is the evaluation level of all fresh produce supply chains, and 〈*a*_*pi*_, *b*_*pi*_〉 represents the value interval of *V*_*pj*_ for *C*_*j*_ indicators under all resilience evaluation levels. The section domain *M*_*p*_ has the following form:

$$M_{p}=\left[\begin{array}{ccc} O_{p,} & C_{j,} & V_{pj} \end{array}\right]=\left[\begin{array}{ccc} O_{p} & c_{1} & v_{p1}\\ \; & c_{2} & v_{p2}\\ \; & \vdots & \vdots \\ \; & c_{n} & v_{pn} \end{array}\right]=\left[\begin{array}{ccc} O_{p} & c_{1} & \langle \begin{array}{cc} a_{p1,} & b_{p1} \end{array}\rangle \\ \; & c_{2} & \langle \begin{array}{cc} a_{p2,} & b_{p2} \end{array}\rangle \\ \; & \vdots & \vdots \\ \; & c_{n} & \langle \begin{array}{cc} a_{pn,} & b_{pn} \end{array}\rangle \end{array}\right]$$

Matter-element to be evaluated: The objects to be evaluated in this paper are the indicators that need to be evaluated for resilience, assuming that *O* is the set of indicators to be evaluated in the indicator layer, *O*_*i*_ represents the resilience level of fresh produce supply chain to be evaluated, *C*_*i*_ represents the resilience evaluation indexes of fresh produce supply chain, and *V*_*i*_ represents the actual measured value of evaluation index *C*_*i*_. The model is as follows:

$$M_{i}=\left[\begin{array}{ccc} O, & C_{i}, & V_{i} \end{array}\right]=\left[\begin{array}{ccc} O_{i} & c_{1} & v_{1}\\ \; & c_{2} & v_{2}\\ \; & \vdots & \vdots \\ \; & c_{n} & v_{n} \end{array}\right]$$

#### 3.3.2. Dependent function

The dependent function is calculated from quantitative data and from an objective point of view to analyse the degree of correlation of the indicator scores on the evaluation intervals, the purpose of which is to respond to the degree of correlation of the resilience indicator scoring data with the various evaluation intervals. The distance between the values of the indicators of the matter-element to be evaluated (resilience indicator) i (i = 1,2,……., m) and the intervals of the classical domain and the section domain is calculated. The specific formula for constructing the dependent function of each indicator is as follows:

$$\rho \left(\begin{array}{cc} v_{i}(e), & V_{oej} \end{array}\right)=\left|\left.v_{i}-\frac{a_{oej}+b_{oej}}{2}\right|\right.-\frac{b_{oej}-a_{oej}}{2}$$

$$\rho \left(\begin{array}{cc} v_{i}(e), & V_{pj} \end{array}\right)=\left|\left.v_{i}-\frac{a_{pj}+b_{pj}}{2}\right|\right.-\frac{b_{pj}-a_{pj}}{2}$$

$$k_{e}\left(v_{i}\right)=\left\{\begin{array}{c} \frac{\rho \left(\begin{array}{cc} v_{i}, & V_{oej} \end{array}\right)}{\rho \left(\begin{array}{cc} v_{i}, & V_{pj} \end{array}\right)-\rho \left(\begin{array}{cc} v_{i}, & V_{oej} \end{array}\right)},\rho \left(\begin{array}{cc} v_{i}, & V_{pj} \end{array}\right)-\rho \left(\begin{array}{cc} v_{i}, & V_{oej} \end{array}\right)\neq 0\\ -\rho \left(\begin{array}{cc} v_{i}, & V_{oej} \end{array}\right)+1,\rho \left(\begin{array}{cc} v_{i}, & V_{pj} \end{array}\right)-\rho \left(\begin{array}{cc} v_{i}, & V_{oej} \end{array}\right)=0 \end{array}\right.$$

#### 3.3.3. Comprehensive dependent function

Comprehensive dependent function is calculated through the weight ratio of each indicator, mainly refers to the degree of attribution of the indicators to be evaluated about each evaluation level, through the value of the comprehensive dependent function and the fresh produce supply chain resilience evaluation level after objective analysis and comparison, from the objective, quantitative expression of the degree of conformity between the conditions and objectives for the subsequent assessment and analysis of the strategy generated to provide quantitative measurement tools. The formula is as follows:

$$k_{e}\left(O\right)=\sum_{i=1}^{m}w_{i}k_{e}\left(v_{i}\right),\sum_{i=1}^{m}w_{i}=1$$

where: *w*_*i*_ is a vector of weights for each evaluation indicator, and the value of the vector of weights is taken to be 1.

This paper adopts the principle of maximum fuzzy affiliation degree, according to the principle of the standard, through the supply chain resilience scientific evaluation and preliminary judgement can be derived:

$$k_{e}\left(O\right)=\text{max}\left\{\left.k_{e}\left(o\right)\right\}\right.\left(e=1,2,\ldots ,s\right)$$

### 3.4. Extension transformation and extension analysis

Extension transformation of extensible analysis mainly tries to apply purposeful and process-oriented extension transformation to the goal and condition basic-element or complex elements in the face of contradictory problems, but before expanding the transformation, it should also consider whether the goal and condition of the problem to be solved can be transformed, and if it can be transformed, then it can find feasible problem-solving channels by opening up a multi-dimensional perspective, which is a method of problem solving and decision making. extensible analysis is a method of problem solving and decision making. extensible analysis is based on the principles of divergence analysis, correlation network, implication system and expandability analysis, and it is used to explore the possibilities of various expandable transformations by replacing, adding, deleting, expanding, reducing, decomposing and copying basic-element or complex elements of the goals and conditions of the problem to be solved in the existence of contradictory problems.

In the extensible analysis method, the principle of dispersion analysis is also known as the divergence tree method. The divergence tree method is based on the principle of dispersion analysis derived from the principle of multiple features of an object and multiple objects of a feature, and it is used to form new basic-elements from the goal or condition of an existing contradictory problem as the entrance to the study, and the divergence analysis expressed in the model is based on the transformations of the object *O*, the nature *C* of the object *O*, or the object *O* with respect to the nature *C* of the quantity and value of the object *V*:

$$\begin{array}{c} \left\{\left.\left(O_{i},C,V_{i}\right),i=1,2,\cdots ,n\right\}\right.\\ \left\{\left.\left(O_{i},C_{i},V\right),i=1,2,\cdots ,n\right\}\right.\\ \left\{\left.\left(O_{i},C,V\right),i=1,2,\cdots ,n\right\}\right. \end{array}\begin{array}{c} ↖\\ \leftarrow \\ ↙ \end{array}\left(O,C,V\right)\begin{array}{c} ↗\\ \to \\ ↘ \end{array}\begin{array}{c} \left\{\left.\left(O,C_{i},V_{i}\right),i=1,2,\cdots ,n\right\}\right.\\ \left\{\left.\left(O,C_{i},V\right),i=1,2,\cdots ,n\right\}\right.\\ \left\{\left.\left(O,C,V_{i}\right),i=1,2,\cdots ,n\right\}\right. \end{array}$$

In the extensible analysis method, the principle of correlation analysis is called the correlation network method. After the formal representation of basic-element for the goals and conditions of the contradictory problem, the correlation network method expands according to the correlation between things, objects and relationships, and the connection between different things constitutes a complex network and when the basic-element of a certain object can be conductive transformation, the transformation of the basic-element leads to the change of other related basic-elements through the correlation of the correlation network, which can get some basic-elements suitable for the solution of the current contradiction problem. basic-elements that are suitable for solving the current contradiction problem. Basic-elements are expressed as follows:

$$M∼\left\{\begin{array}{c} M_{1}∼\left\{\begin{array}{c} M_{11}\\ M_{12}\\ \vdots \\ M_{1n} \end{array}\right.\\ M_{2}∼\left\{\begin{array}{c} M_{21}\\ M_{22}\\ \vdots \\ M_{2n} \end{array}\right.\\ \vdots \\ M_{n}∼\left\{\begin{array}{c} M_{n1}\\ M_{n2}\\ \vdots \\ M_{nn} \end{array}\right. \end{array}\right.$$

Among the extensible analysis methods, the principle of implication analysis is known as the implication system method. In implication analysis, the goal and conditional basic-element A in the contradictory problem are implied in the lower basic-element B. By changing the lower basic-element B, the upper basic-element A will be affected by the lower basic-element B. In other words, if the effective lower basic-element B is realized, then A will also be realized, and this is called the basic-element B implication, which is written as *B* ⇒ *A*, therefore, by implying the system of the goal basic-element and the conditional basic-element in the contradictory problem, we will find the lower basic-element. That performs a multidimensional extensible analysis. The implication system is expressed as follows:

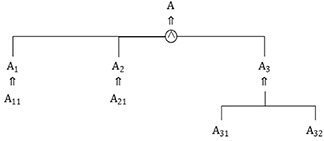


By constructing the primitive model of the above expansion analysis method. We want to better find a feasible way to solve the problem. The basic transformations of extension transformations mainly imply five methods, which are substitution transformation *T*Γ = Γ′, Increase and decrease transformations TΓ_0_ = Γ or *T*_1_Γ = Γ ⊙ Γ_1_, Addition and deletion transformations *T*Γ = αΓ, Subdivision transformation *T*Γ = {Γ_1_, Γ_2_, …, Γ_*n*_} which Γ_1_ ⊕ Γ_2_ ⊕…⊕Γ_*n*_ = Γ, and replication transformation *T*Γ = {Γ, Γ*} In the basic extension transformation method, there exists the transformation *T* that changes Γ_0_ into another object of the same kind Γ multiple objects Γ_1_, Γ_2_,…, Γ_*n*_, and is called *T* as the extension transformation of the object Γ_0_.

## 4. Numerical calculation and analysis of results

### 4.1 Establishment of fresh agricultural products supply chain resilience evaluation index system

In order to establish a scientific and accurate supply chain resilience index system for fresh agricultural products, this paper selects and constructs a supply chain resilience evaluation index system for fresh agricultural products by reviewing relevant literature and other research results and combining the characteristics of the supply chain with the current situation of supply chain resilience of fresh agricultural products and its development potential, following the principles of scientificity, independence and measurability. The whole evaluation index system is divided into four levels: target level, guideline level, indicator level and indicator interpretation. The evaluation of fresh produce supply chain resilience is selected as the target layer, three dimensions of fresh produce resilience, capital resilience and internal resilience are selected as the criterion layer from the whole life cycle of production and operation of fresh produce supply chain, and 13 evaluation indicators are selected as the indicator layer. The evaluation index system for the resilience of the fresh product supply chain is shown in Table 1.

**Table 1:** Fresh produce supply chain resilience evaluation index system.

Target level	Normative layer	Index layer	Interpretation of indicators
Evaluation of Supply Chain Resilience for Fresh Produce	Fresh produce resilience B_1_	Product quality resilience C_1_	Product quality and integrity
		Type of product supply C_2_	Abundance and the need for personalisation
		Product demanded C_3_	Number of customer product requests
		Product supply efficiency C_4_	Order failure rate of products and customer satisfaction of order shipments, etc.
		Product supply time C_5_	Customer product demand response and product delivery time
	Financial resilience B_2_	Financing capacity C_6_	Sustained access to long-term quality capital
		Nodal transport costs C_7_	Costs of trans-shipment, distribution and storage of materials
		maintenance fee C_8_	Maintenance and care costs of vehicles, equipment and information systems for the transport of fresh produce
	Internal resilience B_3_	Human resources protection C_9_	Employee Resource Security, Employee Training Inputs, Employee Training Subjects in the Supply Chain
		Procurement security C_10_	Timeliness and accuracy of fresh produce deliveries
		Production and processing equipment C_11_	Capital investment in fresh produce equipment, equipment production capacity
		logistics support C_12_	Fresh produce transport channels, storage management methods, logistics system reputation, etc.
		Information flow C_13_	Information Acquisition of Demand for Fresh Produce

### 4.2. Calculation of weights for indicators of supply chain resilience for fresh produce

Table 2 below specifies the comparison of quantitative values between indicators.

**Table 2:** Quantitative materiality values.

Factor i compared to factor j	Quantitative value
equal importance	1
slightly important	3
more important	5
high importance	7
utmost importance	9
Intermediate value of two adjacent judgements	2, 4, 6, 8
reciprocal	a_ij_ = 1/a_ji_

Construct the judgment matrix to facilitate the two-by-two comparison of indicators, and then use the expert scoring method to assess the relative importance of each indicator, through the expert scoring will be the indicator two-by-two comparison of the value of the final can be calculated at all levels of indicators of the weight value of *ω* and the judgment matrix of the largest characteristic root of the λmax, and consistency test, when the CR< 0.1 the judgment matrix to meet the consistency. Calculating the weights of the indicators of the fresh produce supply chain lays the foundation for the subsequent calculation of the correlation function and the evaluation of the indicator intervals.

In this paper, a panel of five professionals engaged in as well as having rich theoretical experience in the supply of fresh produce was formed to make comparisons between the indicators through expert scoring, and to make a more scientific guide to the subsequent scoring of the indicators, and the information of the experts is shown in Table 3.

**Table 3:** Anonymous information for experts.

Anonymous experts	Title	Field of study/occupation	Length of time in business
A	Professor	Agricultural supply chain management	11 years
B	Associate professor	Agricultural supply chain management	8 years
C	Agricultural technician	Agricultural information	13 years
D	Associate professor	Agricultural product distribution management	7 years
E	Professor	Agricultural product distribution management	10 years

#### 4.2.1. Calculate the weight of the first level indicators

The judgment matrix for the first level indicators of fresh product resilience B1, financial resilience B2, and internal resilience B3 is shown in Table 4.

**Table 4:** First level indicator judgment matrix.

	B1	B2	B3	ω	λmax	CI	RI	CR	Verdict
**B1**	1	1/2	3	0.3920	3.0194	0.0097	0.52	0.0187<0.1	The judgement matrix satisfies the consistency
**B2**	2	1	4	0.6528					
**B3**	1/3	1/4	1	0.1465					

#### 4.2.2. Calculate the weights of secondary indicators

Construct the weight determination matrix of the secondary indicator system of fresh produce resilience B_1_, product quality resilience C_1_, product supply type C_2_, product being demanded C_3_, product supply efficiency C_4_, product supply time C_5_. The calculation results are shown in Table 5.

**Table 5:** Judgement matrix of secondary indicators of resilience of fresh produce B_1_.

	C_1_	C_2_	C_3_	C_4_	C_5_	ω	λmax	CI	RI	CR	Verdict
**C**_**1**_	1	1/3	3	2	5	0.234	5.1755	0.0877	1.12	0.0783<0.1	The judgement matrix satisfies the consistency
**C**_**2**_	3	1	5	3	7	0.4665					
**C**_**3**_	1/3	1/5	1	1/3	3	0.0859					
**C**_**4**_	1/2	1/3	3	1	4	0.1696					
**C**_**5**_	1/3	1/7	1/3	1/4	1	0.0441					

Construct the weight determination matrix for the secondary indicator system of financial resilience B2, financing capacity C_6_, nodal transport costs C_7_, and maintenance costs C_8_. The calculation results are shown in Table 6.

**Table 6:** Judgement matrix of secondary indicators of financial resilience B_2_.

	C_6_	C_7_	C_8_	ω	λmax	CI	RI	CR	Verdict
**C**_**6**_	1	2	4	0.571	3	0	0.52	0<0.1	The judgement matrix satisfies the consistency
**C**_**7**_	1/2	1	2	0.286					
**C**_**8**_	1/4	1/2	1	0.143					

Construct the weight determination matrix for the secondary indicator system of internal resilience B_3_, human resources security C_9_, procurement security C_10_, production and processing equipment C_11_, logistics security C_12_, and information flow C_13_. The calculation results are shown in Table 7.

**Table 7:** Judgement matrix of secondary indicators of internal resilience B_3_.

	C_9_	C_10_	C_11_	C_12_	C_13_	ω	λmax	CI	RI	CR	Verdict
**C**_**9**_	1	1/7	1/5	1/4	1/6	0.0438	5.1271	0.0318	1.12	0.0284<0.1	The judgement matrix satisfies the consistency
**C**_**10**_	7	1	3	4	2	0.4398					
**C**_**11**_	5	1/3	1	2	1/2	0.1826					
**C**_**12**_	4	1/4	1/2	1	1/3	0.1206					
**C**_**13**_	6	1/2	2	3	1	0.2837					

According to the results of the calculation of the indicators of each indicator layer, the comprehensive weights of the evaluation indicators of the resilience of the supply chain of the production of agricultural products are obtained as shown in Table 8.

**Table 8:** Combined weights of evaluation indicators.

First level indicators		Secondary indicators		Combined weights
norm	weights	norm	weights	
**B**_**1**_	0.392	C_1_	0.234	0.0917
		C_2_	0.467	0.1829
		C_3_	0.086	0.0337
		C_4_	0.17	0.0665
		C_5_	0.044	0.0173
**B**_**2**_	0.653	C_6_	0.571	0.3727
		C_7_	0.286	0.1867
		C_8_	0.143	0.0934
**B**_**3**_	0.147	C_9_	0.044	0.0064
		C_10_	0.44	0.0644
		C_11_	0.183	0.0268
		C_12_	0.121	0.0177
		C_13_	0.284	0.0416

### 4.3. Comprehensive evaluation of extenics

#### 4.3.1. Classical and sectional domains

The classical domain and the section domain determine the classical domain *M*_*oe*_ and the section domain *M*_*p*_ of the indicator, respectively:

$$M_{oe}=\left[\begin{array}{rrrrrr} O_{oe} & O_{1} & O_{2} & O_{3} & O_{4} & O_{5}\\ C_{1} & \left[0,1\right] & \left[1,2\right] & \left[2,3\right] & \left[3,4\right] & \left[4,5\right]\\ C_{2} & \left[0,1\right] & \left[1,2\right] & \left[2,3\right] & \left[3,4\right] & \left[4,5\right]\\ C_{3} & \left[0,1\right] & \left[1,2\right] & \left[2,3\right] & \left[3,4\right] & \left[4,5\right]\\ C_{4} & \left[0,1\right] & \left[1,2\right] & \left[2,3\right] & \left[3,4\right] & \left[4,5\right]\\ C_{5} & \left[0,1\right] & \left[1,2\right] & \left[2,3\right] & \left[3,4\right] & \left[4,5\right]\\ C_{6} & \left[0,1\right] & \left[1,2\right] & \left[2,3\right] & \left[3,4\right] & \left[4,5\right]\\ C_{7} & \left[0,1\right] & \left[1,2\right] & \left[2,3\right] & \left[3,4\right] & \left[4,5\right]\\ C_{8} & \left[0,1\right] & \left[1,2\right] & \left[2,3\right] & \left[3,4\right] & \left[4,5\right]\\ C_{9} & \left[0,1\right] & \left[1,2\right] & \left[2,3\right] & \left[3,4\right] & \left[4,5\right]\\ C_{10} & \left[0,1\right] & \left[1,2\right] & \left[2,3\right] & \left[3,4\right] & \left[4,5\right]\\ C_{11} & \left[0,1\right] & \left[1,2\right] & \left[2,3\right] & \left[3,4\right] & \left[4,5\right]\\ C_{12} & \left[0,1\right] & \left[1,2\right] & \left[2,3\right] & \left[3,4\right] & \left[4,5\right]\\ C_{13} & \left[0,1\right] & \left[1,2\right] & \left[2,3\right] & \left[3,4\right] & \left[4,5\right] \end{array}\right.$$

$$M_{p}=\left[\begin{array}{rrr} O_{p} & C_{1} & \left[0,5\right]\\ \; & C_{2} & \left[0,5\right]\\ \; & C_{3} & \left[0,5\right]\\ \; & C_{4} & \left[0,5\right]\\ \; & C_{5} & \left[0,5\right]\\ \; & C_{6} & \left[0,5\right]\\ \; & C_{7} & \left[0,5\right]\\ \; & C_{8} & \left[0,5\right]\\ \; & C_{9} & \left[0,5\right]\\ \; & C_{10} & \left[0,5\right]\\ \; & C_{11} & \left[0,5\right]\\ \; & C_{12} & \left[0,5\right]\\ \; & C_{13} & \left[0,5\right] \end{array}\right]$$

The classical extension domain divides the evaluation of fresh produce supply chain resilience indicators into 5 quantitative value intervals, when an indicator C_i_ is in the interval [0, 1], it means the indicator is very poor, in the interval [1, 2], it means the indicator is poor, in the interval [2, 3], it means the indicator is ordinary, in the interval [3, 4], it means the indicator is good, and in the interval [4, 5], it means the indicator is excellent. The [0, 5] of the section field represents the range of values that the indicator can take.

#### 4.3.2. Construct matter-element to be evaluated

This paper takes fresh agricultural products supply chain enterprise M as the object, according to the actual operation of the supply chain of the enterprise M to be evaluated, invites experts to score according to the scoring standard by using the qualitative questionnaire, and carries out the preliminary evaluation of the scores of each index of the supply chain status of the fresh agricultural products enterprise through expert evaluation and other ways, and can get the matter-element to be evaluated:

$$M=\left[\begin{array}{rrr} O & C_{1} & 3.20\\ \; & C_{2} & 2.95\\ \; & C_{3} & 3.10\\ \; & C_{4} & 2.40\\ \; & C_{5} & 3.25\\ \; & C_{6} & 3.20\\ \; & C_{7} & 4.10\\ \; & C_{8} & 3.80\\ \; & C_{9} & 3.15\\ \; & C_{10} & 4.15\\ \; & C_{11} & 3.00\\ \; & C_{12} & 2.06\\ \; & C_{13} & 4.28 \end{array}\right]$$

According to the dependent function formula and the comprehensive dependent function formula, the second-level indicator *C*_j_ of the fresh produce supply chain resilience evaluation can calculate the correlation degree about the *O*_1_ level. Take indicator C_1_ in the matter-element to be evaluated of enterprise M as an example:

$$\rho \left(\begin{array}{cc} v_{1}, & V_{o11} \end{array}\right)=\left|\left.3.20-\frac{0+1}{2}\right|\right.-\frac{1-0}{2}=2.2$$

$$\rho \left(\begin{array}{cc} v_{1}, & V_{p1} \end{array}\right)=\left|\left.3.20-\frac{0+5}{2}\right|\right.-\frac{5-0}{2}=-1.8$$

Since2.2- (-1.8) ≠0, so:

$$k_{1}(v_{1})=\frac{\rho \left(\begin{array}{cc} v_{1}, & V_{o11} \end{array}\right)}{\rho \left(\begin{array}{cc} v_{1}, & V_{p1} \end{array}\right)-\rho \left(\begin{array}{cc} v_{1}, & V_{o11} \end{array}\right)}=\frac{2.2}{-1.8-2.2}=-0.55$$

Similarly, the order of correlation of the indicators of firm M with respect to the *O*_1_ level are (-0.5500, -0.4875, -0.5250, -0.3500, -0.5625, -0.5500, -0.775, -0.7000, -0.5375, -0.7875, -0.5000, -0.3398, -0.8200). The value of the composite dependent function k_1_ for firm M with respect to evaluation level e = 1 is calculated as:

$$K_{1}=\sum_{i=1}^{13}\omega_{i}k_{1}(v_{i})=-0.7153$$

The order of correlation of the indicators of firm M with respect to the *O*_2_ level are(-0.3750, -0.3167, -0.3667, -0.1333, -0.4167, -0.4000, -0.6610, -0.6000, -0.3833, -0.7167, -0.3333, -0.0283, -0.7600). The value of the composite dependent function k_2_ for firm M with respect to evaluation level e = 2 is calculated as:

$$K_{2}=\sum_{i=1}^{13}\omega_{i}k_{2}(v_{i})=-0.5389$$

The order of correlation of the indicators of firm M with respect to the *O*_3_ level are (-0.1000, -0.0250, -0.0333, 0.1429, -0.0833, -0.0667, -0.5500, -0.4000, -0.0500, -0.5750, 0, 0.0283, -0.6400). The value of the composite dependent function k_3_ for firm M with respect to evaluation level e = 3 is calculated as:

$$K_{3}=\sum_{i=1}^{13}\omega_{i}k_{3}(v_{i})=-0.235$$

The order of correlation of the indicators of firm M with respect to the *O*_4_ level are (0.1250, -0.0250, 0.0500, 0.2000, 0.1667, 0.1000, -0.1000, 0.4000, 0.0150, -0.1500, 0, -0.3133, -0.2800). The value of the composite dependent function k_4_ for firm M with respect to evaluation level e = 4 is calculated as:

$$K_{4}=\sum_{i=1}^{13}\omega_{i}k_{4}(v_{i})=0.0335$$

The order of correlation of the indicators of firm M with respect to the *O*_5_ level are (-0.3077, -0.5926, -0.3214, -0.8000, -0.3000, -0.2800, 0.1000, -0.3684, -0.3148, 0.1250, -0.3333, -0.4850, 0.2800). The value of the composite dependent function k_5_ for firm M with respect to evaluation level e = 5 is calculated as:

$$K_{5}=\sum_{i=1}^{13}\omega_{i}k_{5}(v_{i})=-0.326$$

Analysing the value of the comprehensive evaluation dependent function for more levels of enterprise M, it is concluded that regarding the evaluation interval evaluation level e = 4, the value of the comprehensive dependent function at this time is positive, indicating that the resilience evaluation result of this fresh produce supply chain enterprise M is good. Regarding the evaluation level e = 5, only two resilience evaluation indexes are positive, indicating that only two indexes are in the excellent state. Through the overall analysis, although the comprehensive tenacity score of this enterprise has reached the good interval, many indexes still have a lot of room for improvement to reach the excellent interval.

### 4.4. Modelling incompatible problems of fresh agricultural products

#### 4.4.1. Constructing the incompatible problem model

From the analysis of the dependent function of the indicators of each evaluation level, in the resilience evaluation results of enterprise M, the product supply type C2, product supply efficiency C4 and logistics security C12 in the evaluation level e = 3 when the dependent function value is positive, which indicates that these evaluation indicators scores are located in the ordinary interval, the evaluation object of enterprise M needs to optimise the indicators, so as to arrive at the enterprise’s resilience of the overall score interval, and then strive to punch the excellent. Then strive to punch excellent.

Product quality resilience C1, product demand C3, product supply time C5, financing capacity C6, maintenance fee C8, human resources security C9. These resilience indicators have a positive dependent function value at evaluation level e = 4, which indicates that the scores of these evaluation indicators are in the good range, proving that these indicators have a lot of potential for development, and they are the reserve force of the excellent indicators, which can be used for the transformation of enterprise M into a high resilience fresh produce supply chain enterprise. high resilience fresh produce supply chain enterprise. Production and processing equipment C11 has a dependent function value of 0 at evaluation levels e = 3 and e = 4, which indicates that the indicator is at the boundary of the two evaluation levels, and there is still room for improvement of the indicator through the analysis of the specific score of the indicator.


$$\begin{array}{c} P=G*L=\\ \left[\begin{array}{rrr} \text{Elevation}, & Dominating\;object, & \text{Supply}\;\text{chain}\;\text{resilience}\\ \; & Acting\;object, & \text{Enterprise}\;\text{M} \end{array}\right]\\ {}*\left[\begin{array}{rrr} \text{Resilience}\;\text{factors}, & \text{Source}, & \text{Configuration}\;\text{of}\;\text{the}\;\text{enterprise}\\ \; & \text{Rating}, & \text{Low} \end{array}\right] \end{array}$$

The goal is to improve the supply chain resilience of fresh produce firm M. When the goal is constant, we make the incompatible problem dissolve by changing the conditions of the problem.

#### 4.4.2. Establishment of core problem

According to the dependent function value of supply chain resilience evaluation and each index score for the selection of indicators to carry out the establishment of the nuclear problem, this paper selects the lower score has a lot of room for improvement of a few indicators, the product supply type C_2_, the product is demanded C_3_, the product supply efficiency C_4_, human resources security C_9_, the production and processing equipment C_11_, the logistics security C_12_ is in the ordinary or good grade range, some of these indicators still have a lot of room for improvement, enterprise M wants to do supply chain resilience improvement, it is necessary to optimise the indicators. Some of these indicators still have a lot of room for improvement, and Enterprise M needs to optimise the indicators if it wants to achieve supply chain resilience.

The above matter-element model is constructed based on the values taken for the evaluation of the resilience indicators of the firm M:

$$M=\left[\begin{array}{rrr} \text{Resilience}, & Type\;of\;product\;supply, & 2.95\\ \; & Product\;demanded, & 3.10\\ \; & Supply\;efficiency, & 2.40\\ \; & \text{Human}\;\text{resources}\;\text{protection}, & 3.15\\ \; & Production\;and\;processing\;equipment, & 3.00\\ \; & \text{logistics}\;\text{support}, & 2.06 \end{array}\right]$$

In this resilience enhancement strategy, the resilience of fresh produce supply chain enterprise M is the object, the indicators of the above enterprise M are the characteristics, and their evaluation scores are the quantitative values in the basic-element. Expression model of the core problem constructed to solve the paradoxical problem when the objective is not transformed:

$$p_{0}=g_{0}*l_{0}=\left[\begin{array}{rrr} \text{Elevation}, & Dominating\;object, & \text{Resilience}\\ \; & Type\;of\;product\;supply, & \text{Richness}\\ \; & Product\;demanded, & \text{stockpile}\\ \; & Supply\;efficiency, & \text{Level}\\ \; & \text{Human}\;\text{resources}\;\text{protection,} & \text{Quantities}\\ \; & Production\;and\;processing\;equipment, & \text{Degree}\\ \; & \text{logistics}\;\text{support}, & \text{Abilities} \end{array}\right]$$

$$l_{0}=\left[l_{01}\wedge l_{02}\wedge l_{03}\wedge l_{04}\wedge l_{05}\wedge l_{06}\right]$$

The conditional matter-element is:

$$\begin{array}{c} L=\left[\begin{array}{ccc} Type\;of\;product\;supply, & \text{Number}\;\text{of}\;\text{individuals}, & 2.95 \end{array}\right]\wedge \left[\begin{array}{ccc} Product\;demanded, & \text{Available}\;\text{quantity}, & 3.10 \end{array}\right]\\ \wedge \left[\begin{array}{ccc} Supply\;efficiency, & \text{Efficiency}\;\text{value,} & 2.40 \end{array}\right]\wedge \left[\begin{array}{ccc} \text{Human}\;\text{resources}\;\text{protection,} & \text{Quantities,} & 3.15 \end{array}\right]\\ \wedge \left[\begin{array}{ccc} Production\;and\;processing\;equipment, & \text{Degree}, & 3.00 \end{array}\right]\wedge \left[\begin{array}{ccc} \text{Logistics}\;\text{support}, & \text{Abilities}, & 2.06 \end{array}\right] \end{array}$$

In order to achieve the goal of the enterprise M resilience to improve the nuclear problem, we need to combine the reality of the enterprise and the weight of the indicator table to divide the classical domain of each condition base element, the expert group to analyse and formulate the goal of the indicator to improve the classical domain of the target interval, which are divided into the nuclear problem of the condition base element of the target classical domain of the target interval: the type of supply of the target classical domain of the product interval for the (3.5, 4), the product is demanded for the target classical domain of the interval for the (3.5, 4.2), product supply efficiency objective classical domain interval is (3.2, 4), human resources security objective classical domain interval is (3.6, 4.3), production and processing equipment (3.5, 4.5), logistics security objective classical domain interval is (3.2, 4).

From the perspective of enterprise development strategy and objectives, as the long as the target of resilience improvement can be achieved as scheduled, less the input of elements is the better for the enterprise,.therefore, the conditional indicator to construct the characteristics of each optimal point xo is located in the left side of the classical domain interval *X* = 〈*a*_*oej*_, *b*_*oej*_〉 that is the optimal solution. *x*_0_ = *a* The dependent function of *x*_0_ = *a* is as follows:

$$k\left(x\right)=\left\{\begin{array}{cc} \frac{x-a}{b-a} & x<a\\ \frac{b-x}{b-a} & x\geq a \end{array}\right.$$

The compatibility function of the present indicator scores with the classical domain of their respective targets can be obtained from the above equations as: k(x_1_) = -1.1, k(x_2_) = -0.5714, k(x_3_) = -1, k(x_4_) = -0.6429, k(x_5_) = -0.5, k(x_6_) = -1.425.

Constructing the total compatibility function of objectives and conditions in core problems:

$$K\left(p_{0}\right)=\overset{6}{\underset{i=1}{\wedge }}k\left(v_{i}\right)=k\left(v\left(l_{1}\right)\right)\wedge k\left(v\left(l_{2}\right)\right)\wedge k\left(v\left(l_{3}\right)\right)\wedge k\left(v\left(l_{4}\right)\right)\wedge k\left(v\left(l_{5}\right)\right)\wedge k\left(v\left(l_{6}\right)\right)$$

Let *k*(*x*_*i*_) be the dependent function of the conditional basic-element *l*_*i*_, and *x*_*i*_ be the value of the feature evaluation of the basic-element condition *l*_*i*_. If we want the compatibility function *K*(*p*_0_) of the overall index to be >0, we need to solve the problem of each conditional primitive to optimize it so that the compatibility function *k*(*x*_*i*_) will be >0. However, at this time, the total compatibility function is:

$$\begin{array}{c} K\left(p_{0}\right)=\overset{6}{\underset{i=1}{\wedge }}k\left(v_{i}\right)=k\left(v\left(l_{1}\right)\right)\wedge k\left(v\left(l_{2}\right)\right)\wedge k\left(v\left(l_{3}\right)\right)\wedge k\left(v\left(l_{5}\right)\right)\wedge k\left(v\left(l_{6}\right)\right)\wedge k\left(v\left(l_{6}\right)\right)\\ =-1.1\wedge -0.5714\wedge -1\wedge -0.6429\wedge -0.5\wedge -1.425<0 \end{array}$$

The total compatibility function of the core problem at this point *k*(*p*) < 0, we need to perform extension and subdivision transformations of the conditional basic-elements of the core problem to solve the incompatibility problem at this point.

### 4.5. Extension and subdivision transformations

In order to enhance the M resilience of fresh produce supply chain enterprises to solve the incompatible problem, firstly, the extensible analysis method is used to analyse the complex relationship network of conditional basic-elements in a scalable way, so as to construct the correlation network of conditional basic-elements, and then, the "leaves" on the branch trunks in the correlation network are analysed by dispersion analysis, and the dispersed basic-elements are subjected to scalable transformations, so that the basic-elements of the strategies that can enhance the resilience of the supply chain can be generated eventually.

By searching the database of supply chain resilience elements, correlation analysis was conducted to find out the relevant elements that have a positive influence on each condition element, and to find out the types of product supply *l*_01_ related to product development *l*_011_ and market information collection *l*_012_.

Divergence analysis of product development *l*_01_ obtained by correlation analysis *l*_011_ product supply types of conditional basic-elements, and market information collection *l*_012_:

$${\text{l}}_{01}∼\left\{\begin{array}{c} {\text{l}}_{011}=\left[\begin{array}{rrr} \text{Product}\;\text{development}, & \text{Executing}\;\text{entity}, & \text{Enterprise}\;\text{M}\\ \; & \text{Source}\;\text{of}\;\text{funds}, & \text{Enterprise}\;\text{capital}\\ \; & \text{Investment}\;\text{amount}, & {\text{v}}_{011} \end{array}\right]\\ {\text{l}}_{012}=\left[\begin{array}{rrr} \text{Collecting}, & \text{Dominating}\;\text{object}, & \text{market}\;\text{information}\\ \; & \text{Acting}\;\text{object}, & \text{Enterprise}\;\text{M}\;\text{employees}\\ \; & \text{informativeness}, & {\text{v}}_{012} \end{array}\right] \end{array}\right.$$

In order to achieve the goal of solving the incompatibility problem, it is necessary to implement extension transformations on the conditioned basic-elements *l*_01_ dispersed from *l*_011_ and *l*_012_ conditioned basic-elements, applying the methods in the method of extension transformations to carry out a variety of transformations on the dispersed basic-elements, dispersed basic-elements have relevance, eventually the initial conditioned basic-elements will be changed through the conductivity of things, existence of (*T*_011_ ˄ *T*_012_) ⇒ *T*_01_, (*T*_011_ ˅ *T*_012_) ⇒ *T*_01_, and the conditioned basic-elements *l*_01_ of the relevance *l*_011_ and *l*_012_ of extension transformations as a substitution method, the basic-element model is as follows:

$$T_{011}l_{011}=\left\{\begin{array}{r} T_{011}^{\prime }l_{011}=l_{011}^{\prime }=\left[\begin{array}{rrr} \text{Product}\;\text{development}, & \text{Executing}\;\text{entity}, & \text{Enterprise}\;\text{M}\\ \; & \text{Source}\;\text{of}\;\text{funds}, & \text{Bank}\;\text{Loans}\\ \; & \text{Investment}\;\text{amount}, & v_{011}^{\prime } \end{array}\right]\\ T_{011}^{\prime \prime }l_{011}=l_{011}^{\prime \prime }=\left[\begin{array}{rrr} \text{Product}\;\text{development}, & \text{Executing}\;\text{entity}, & \text{Enterprise}\;\text{M}\\ \; & \text{Source}\;\text{of}\;\text{funds}, & \text{Government}\;\text{subsidies}\\ \; & \text{Investment}\;\text{amount}, & v_{011}^{\prime \prime } \end{array},\right]\\ \begin{array}{l} T_{011}^{\prime \prime \prime }l_{011}=l_{011}^{\prime \prime \prime }=\left[\begin{array}{rrr} \text{Product}\;\text{development}, & \text{Executing}\;\text{entity}, & \text{Enterprise}\;\text{M}\\ \; & \text{Source}\;\text{of}\;\text{funds}, & \text{investor}\;\text{capital}\\ \; & \text{Investment}\;\text{amount}, & v_{011}^{\prime \prime \prime } \end{array}\right]\\ \cdots \end{array}\\ \; \end{array}\right.$$

$${\text{T}}_{012}{\text{l}}_{012}=\left\{\begin{array}{c} \begin{array}{l} {\text{T}}_{012}^{\prime }{\text{l}}_{012}={\text{l}}_{012}^{\prime }={\text{l}}_{0121}\left[\begin{array}{rrr} \text{Collecting}, & \text{Dominating}\;\text{object}, & \text{market}\;\text{information}\\ \; & \text{Acting}\;\text{object}, & \text{Professional}\;\text{Information}\;\text{Enterprise}\\ \; & \text{informativeness}, & {\text{v}}_{0121}^{\prime } \end{array}\right]\\ {\text{T}}_{012}^{\prime \prime }{\text{l}}_{012}={\text{l}}_{012}^{\prime \prime }={\text{l}}_{0122}\left[\begin{array}{rrr} \text{Collecting}, & \text{Dominating}\;\text{object}, & \text{market}\;\text{information}\\ \; & \text{Acting}\;\text{object}, & \text{Intelligent}\;\text{platforms}\\ \; & \text{informativeness}, & {\text{v}}_{0122}^{\prime } \end{array}\right]\\ \cdots \end{array}\\ \; \end{array}\right.$$

Combining and transforming the new conditional basic-elements produced by extension transformations:

$$T_{02}l_{01}=\left\{\begin{array}{lll} l_{01}^{\prime }=T_{01}^{\prime }l_{01} & ⇐ & \{T_{021}^{\prime }l_{021}\wedge T_{012}^{\prime }l_{022}\\ l_{01}^{\prime \prime }=T_{01}^{\prime \prime }l_{01} & ⇐ & \{T_{021}^{\prime \prime }l_{021}\wedge T_{012}^{\prime }l_{022}\\ l_{01}^{\prime \prime \prime }=T_{01}^{\prime \prime \prime }l_{01} & ⇐ & \{T_{021}^{\prime \prime \prime }l_{021}\wedge T_{012}^{\prime }l_{022}\\ l_{01}^{\prime \prime \prime \prime }=T_{01}^{\prime \prime \prime \prime }l_{01} & ⇐ & \{T_{021}^{\prime }l_{021}\wedge T_{022}^{\prime \prime }l_{022}\\ l_{01}^{\prime \prime \prime \prime \prime }=T_{01}^{\prime \prime \prime \prime \prime }l_{01} & ⇐ & \{T_{021}^{\prime \prime }l_{021}\wedge T_{012}^{\prime \prime }l_{022}\\ l_{01}^{\prime \prime \prime \prime \prime \prime }=T_{01}^{\prime \prime \prime \prime \prime \prime }l_{01} & ⇐ & \{T_{021}^{\prime \prime \prime }l_{021}\wedge T_{012}^{\prime \prime }l_{022}\\ \cdots & \; & \; \end{array}\right.$$

In this case, if there exists the dependent function *k*(*v*(*T*_01_*l*_01_)) ≥ 0 of the conditional basic-elements after the extension transformation, then it means that the transformation is feasible.

Divergence analysis is performed on *l*_021_ sales strategy and *l*_022_ inventory management obtained by analyzing the conditional basic-elements *l*_02_ product demand correlation:

$$l_{02}∼\left\{\begin{array}{l} l_{021}=\left[\begin{array}{rrr} \text{Sales}\;\text{strategy}, & \text{Mode,} & \text{Enterprise}\;\text{M}\\ \; & \text{Sales}\;\text{volume,} & v_{021} \end{array}\right]\\ l_{022}=\left[\begin{array}{rrr} \text{Inventory}\;\text{management,} & \text{Mode,} & \text{Reorder}\;\text{point}\;\text{inventory}\;\text{method}\\ \; & \text{Stockpile,} & v_{022} \end{array}\right] \end{array}\right.$$

Similarly, the associated basic-elements *l*_021_ and *l*_022_ of *l*_02_ can be extension transformed as:

$$T_{021}l_{021}\left\{\begin{array}{c} T_{021}^{\prime }l_{021}=l_{021}^{\prime }=\left[\begin{array}{rrr} \text{Sales}\;\text{strategy}, & \text{Mode}, & \text{Media}\;\text{communication}\\ \; & \text{Sales}\;\text{volume}, & v_{021}^{\prime } \end{array}\right]\\ T_{021}^{\prime \prime }l_{021}=l_{021}^{\prime \prime }=\left[\begin{array}{rrr} \text{Sales}\;\text{strategy}, & \text{Mode}, & \text{Word-of-mouth}\;\text{communication}\\ \; & \text{Sales}\;\text{volume}, & v_{021}^{\prime \prime } \end{array}\right]\\ \begin{array}{l} T_{021}^{\prime \prime \prime }l_{021}=l_{021}^{\prime \prime \prime }=\left[\begin{array}{rrr} \text{Sales}\;\text{strategy}, & \text{Mode}, & \text{Network}\;\text{event}\;\text{promotion}\\ \; & \text{Sales}\;\text{volume}, & v_{021}^{\prime \prime \prime } \end{array}\right]\\ \cdots \end{array} \end{array}\right.$$

$$T_{022}l_{022}\left\{\begin{array}{c} T_{022}^{\prime }l_{022}=l_{022}^{\prime }=\left[\begin{array}{rrr} \text{Inventory}\;\text{management}, & \text{Mode}, & \text{Economic}\;\text{Volume}\;\text{Method}\\ \; & \text{Stockpile}, & v_{022}^{\prime } \end{array}\right]\\ T_{022}^{\prime \prime }l_{022}=l_{022}^{\prime \prime }=\left[\begin{array}{rrr} \text{Inventory}\;\text{management}, & \text{Mode}, & \text{Just-in-time}\;\text{inventory}\;\text{management}\;\text{method}\\ \; & \text{Stockpile}, & v_{022}^{\prime \prime } \end{array}\right]\\ \begin{array}{l} T_{022}^{\prime \prime \prime }l_{022}=l_{022}^{\prime \prime \prime }=\left[\begin{array}{rrr} \text{Inventory}\;\text{management}, & \text{Mode}, & \text{ABC}\;\text{Focused}\;\text{Control}\;\text{Method}\\ \; & \text{Stockpile}, & v_{022}^{\prime \prime \prime } \end{array}\right]\\ \cdots \end{array} \end{array}\right.$$

Combining and transforming the new conditional basic-elements produced by extension transformations:

$$T_{02}l_{02}=\left\{\begin{array}{l} l_{02}^{\prime }=T_{02}^{\prime }l_{02}⇐\{T_{021}^{\prime }l_{021}\wedge T_{012}^{\prime }l_{022}\\ l_{02}^{\prime \prime }=T_{02}^{\prime \prime }l_{02}⇐\{T_{021}^{\prime \prime }l_{021}\wedge T_{012}^{\prime }l_{022}\\ l_{02}^{\prime \prime \prime }=T_{02}^{\prime \prime \prime }l_{02}⇐\{T_{021}^{\prime \prime \prime }l_{021}\wedge T_{012}^{\prime }l_{022}\\ l_{02}^{\prime \prime \prime \prime }=T_{02}^{\prime \prime \prime \prime }l_{02}⇐\{T_{021}^{\prime }l_{021}\wedge T_{022}^{\prime \prime }l_{022}\\ l_{02}^{\prime \prime \prime \prime \prime }=T_{02}^{\prime \prime \prime \prime \prime }l_{02}⇐\{T_{021}^{\prime \prime }l_{021}\wedge T_{012}^{\prime \prime }l_{022}\\ l_{02}^{\prime \prime \prime \prime \prime \prime }=T_{02}^{\prime \prime \prime \prime \prime \prime }l_{02}⇐\{T_{021}^{\prime \prime \prime }l_{021}\wedge T_{012}^{\prime \prime }l_{022}\\ \cdots \end{array}\right.$$

In this case, if there exists the dependent function *k*(*v*(*T*_02_*l*_02_)) ≥ 0 of the conditional basic-elements after the extension transformation, then it means that the transformation is feasible.

Divergence analysis of *l*_031_ goods transport and *l*_032_ order security obtained from the analysis of the *l*_03_ product supply chain efficiency correlation of the conditional basic-elements:

$$l_{03}∼\left\{\begin{array}{c} l_{031}=\left[\begin{array}{rrr} \text{Transported}, & Dominating\;object, & \text{Cargoes}\\ \; & \text{Carrier}, & \text{Manual}\;\text{mini-transporter}\\ \; & \text{Volume}\;\text{of}\;\text{freight}, & v_{031} \end{array}\right]\\ l_{032}=\left[\begin{array}{rrr} \text{Guarantee}, & Dominating\;object, & \text{Orders}\\ \; & \text{Methodology}, & \text{Contract}\\ \; & \text{Missing}\;\text{order}\;\text{rate}, & v_{032} \end{array}\right] \end{array}\right.$$

Similarly, the associated basic-elements *l*_031_ and *l*_032_ of *l*_03_ can be extension transformed as:

$${\text{T}}_{031}{\text{l}}_{031}\left\{\begin{array}{c} {\text{T}}_{031}^{\prime }{\text{l}}_{031}={\text{l}}_{031}^{\prime }=\left[\begin{array}{rrr} \text{Transported,} & \text{Dominating}\;\text{object}, & \text{Cargo}\\ \; & \text{Carrier,} & \text{Intelligent}\;\text{unmanned}\;\text{transport}\;\text{vehicle}\\ \; & \text{Volume}\;\text{of}\;\text{freight}, & {\text{v}}_{031}^{\prime } \end{array}\right]\\ \begin{array}{l} {\text{T}}_{031}^{\prime \prime }{\text{l}}_{031}={\text{l}}_{031}^{\prime \prime }=\left[\begin{array}{rrr} \text{Transported}, & \text{Dominating}\;\text{object}, & \text{Cargo}\\ \; & \text{Carrier}, & \text{Specialist}\;\text{manual}\;\text{transport}\;\text{vehicle}\\ \; & \text{Volume}\;\text{of}\;\text{freight}, & {\text{v}}_{031}^{\prime \prime } \end{array}\right]\\ \cdots \end{array} \end{array}\right.$$

$${\text{T}}_{032}{\text{l}}_{032}\left\{\begin{array}{c} {\text{T}}_{032}^{\prime }{\text{l}}_{032}={\text{l}}_{032}^{\prime }=\left[\begin{array}{rrr} \text{Guarantee}, & \text{Dominating}\;\text{object}, & \text{Order}\\ \; & \text{Methodology}, & \text{Improving}\;\text{customer}\;\text{service}\\ \; & \text{Missing}\;\text{order}\;\text{rate}, & {\text{v}}_{032}^{\prime } \end{array}\right]\\ \begin{array}{l} {\text{T}}_{032}^{\prime \prime }{\text{l}}_{032}={\text{l}}_{032}^{\prime \prime }=\left[\begin{array}{rrr} \text{Guarantee}, & \text{Dominating}\;\text{object}, & \text{Order}\\ \; & \text{Methodology}, & \text{Enhanced}\;\text{order}\;\text{process}\\ \; & \text{Missing}\;\text{order}\;\text{rate}, & {\text{v}}_{032}^{\prime \prime } \end{array}\right]\\ \cdots \end{array} \end{array}\right.$$

Combining and transforming the new conditional basic-elements produced by extension transformations:

$$T_{03}l_{03}=\left\{\begin{array}{lll} l_{03}^{\prime }=T_{03}^{\prime }l_{03} & ⇐ & \{T_{031}^{\prime }l_{031}\wedge T_{032}^{\prime }l_{032}\\ l_{03}^{\prime \prime }=T_{03}^{\prime \prime }l_{03} & ⇐ & \{T_{031}^{\prime \prime }l_{031}\wedge T_{032}^{\prime }l_{032}\\ l_{03}^{\prime \prime \prime }=T_{03}^{\prime \prime \prime }l_{03} & ⇐ & \{T_{031}^{\prime }l_{031}\wedge T_{032}^{\prime \prime }l_{032}\\ l_{03}^{\prime \prime \prime \prime }=T_{03}^{\prime \prime \prime \prime }l_{03} & ⇐ & \{T_{031}^{\prime \prime }l_{031}\wedge T_{032}^{\prime \prime }l_{032}\\ \ldots & \; & \; \end{array}\right.$$

In this case, if there exists the dependent function *k*(*v*(*T*_03_*l*_03_)) ≥ 0 of the conditional basic-elements after the extension transformation, then it means that the transformation is feasible.

Divergence analysis of *l*_041_ training methods obtained from the *l*_04_ human resource security correlation analysis of the conditional basic-elements:

$${\text{l}}_{04}∼{\text{l}}_{041}=\left[\begin{array}{rrr} \text{Training}, & \text{Dominating}\;\text{object}, & \text{Enterprise}\;\text{M}\;\text{employees}\\ \; & \text{Training}\;\text{method}, & \text{Traditional}\;\text{face-to-face}\;\text{training}\\ \; & \text{Uptake}, & \text{Ordinary} \end{array}\right]$$

Similarly, the associated basic-elements *l*_041_ of *l*_04_ can be extension transformed as:

$${\text{T}}_{041}{\text{l}}_{041}\left\{\begin{array}{l} {\text{T}}_{041}^{\prime }{\text{l}}_{041}={\text{l}}_{041}^{\prime }=\left[\begin{array}{rrr} \text{Training}, & \text{Dominating}\;\text{object}, & \text{Enterprise}\;\text{M}\;\text{employees}\\ \; & \text{Training}\;\text{method}, & \text{Online}\;\text{web-based}\;\text{training}\\ \; & \text{Uptake}, & {\text{v}}_{041}^{\prime } \end{array}\right]\\ {\text{T}}_{041}^{\prime \prime }{\text{l}}_{041}={\text{l}}_{041}^{\prime \prime }=\left[\begin{array}{rrr} \text{Training}, & \text{Dominating}\;\text{object}, & \text{Enterprise}\;\text{M}\;\text{employees}\\ \; & \text{Training}\;\text{method}, & \text{Blended}\;\text{training}\\ \; & \text{Uptake}, & {\text{v}}_{041}^{\prime \prime } \end{array}\right]\\ {\text{T}}_{041}^{\prime \prime \prime }{\text{l}}_{041}={\text{l}}_{041}^{\prime \prime \prime }=\left[\begin{array}{rrr} \text{Training}, & \text{Dominating}\;\text{object}, & \text{Enterprise}\;\text{M}\;\text{employees}\\ \; & \text{Training}\;\text{method}, & \text{self-directed}\;\text{learning}\\ \; & \text{Uptake,} & {\text{v}}_{041}^{\prime \prime \prime } \end{array}\right]\\ {\text{T}}_{041}^{\prime \prime \prime \prime }{\text{l}}_{041}={\text{l}}_{041}^{\prime \prime \prime \prime }={\text{l}}_{041}^{\prime }⊕{\text{l}}_{041}^{\prime \prime }=\left[\begin{array}{rrr} \text{Training}, & \text{Dominating}\;\text{object}, & \text{Enterprise}\;\text{M}\;\text{employees}\\ \; & \text{Training}\;\text{method}, & \text{Online}\;\text{web-based}\;\text{training}⊕\text{Blended}\;\text{training}\\ \; & \text{Uptake}, & {\text{v}}_{041}^{\prime \prime \prime \prime } \end{array}\right]\\ {\text{T}}_{041}^{\prime \prime \prime \prime \prime }{\text{l}}_{041}={\text{l}}_{041}^{\prime \prime \prime \prime \prime }={\text{l}}_{041}^{\prime }⊕{\text{l}}_{041}^{\prime \prime \prime }=\left[\begin{array}{rrr} \text{Training}, & \text{Dominating}\;\text{object}, & \text{Enterprise}\;\text{M}\;\text{employees}\\ \; & \text{Training}\;\text{method}, & \text{Online}\;\text{web-based}\;\text{training}⊕\text{self-directed}\;\text{learning}\\ \; & \text{Uptake}, & {\text{v}}_{041}^{\prime \prime \prime \prime \prime } \end{array}\right]\\ {\text{T}}_{041}^{\prime \prime \prime \prime \prime \prime }{\text{l}}_{041}={\text{l}}_{041}^{\prime \prime \prime \prime \prime \prime }={\text{l}}_{041}^{\prime \prime }⊕{\text{l}}_{041}^{\prime \prime \prime }=\left[\begin{array}{rrr} \text{Training}, & \text{Dominating}\;\text{object}, & \text{Enterprise}\;\text{M}\;\text{employees}\\ \; & \text{Training}\;\text{method}, & \text{Blended}\;\text{training}⊕\text{self-directed}\;\text{learning}\\ \; & \text{Uptake}, & {\text{v}}_{041}^{\prime \prime \prime \prime \prime \prime } \end{array}\right]\\ {\text{T}}_{041}^{\prime \prime \prime \prime \prime \prime \prime }{\text{l}}_{041}={\text{l}}_{041}^{\prime \prime \prime \prime \prime \prime \prime }={\text{l}}_{041}^{\prime }⊕{\text{l}}_{041}^{\prime \prime }⊕{\text{l}}_{041}^{\prime \prime \prime }=\left[,\begin{array}{rrr} \text{Training}, & \text{Dominating}\;\text{object}, & \text{Enterprise}\;\text{M}\;\text{employees}\\ \; & \text{Training}\;\text{method}, & \text{Online}\;\text{web-based}\;\text{training}⊕\text{Blended}\;\text{training}⊕\text{self-directed}\;\text{learning}\\ \; & \text{Uptake,} & {\text{v}}_{041}^{\prime \prime \prime \prime } \end{array}\right]\\ \cdots \end{array}\right.$$

By the conductive nature of transformations, a conductive transformation (*T*_*m*_ ˄ *T*_*n*_ ⋯˄ *T*_*i*_ ⋯) ⇒ *T*_04_ occurs such that $T_{04}l_{041}=l_{04}^{\prime }$. ln this case, if there exists the dependent function *k*(*v*(*T*_04_*l*_04_)) ≥ 0 of the conditional basic-elements after the extension transformation, then it means that the transformation is feasible.

Divergence analysis of *l*_051_ production equipment inputs obtained from the *l*_05_ production and processing equipment correlation analysis of the conditional basic-elements:

$$l_{05}∼l_{051}=\left[\begin{array}{rrr} \text{Capital}\;\text{investment}\;\text{in}\;\text{equipment}, & \text{Executing}\;\text{entity}, & \text{Enterprise}\;\text{M}\\ \; & \text{Typology,} & \text{Artificial}\;\text{traditional}\;\text{production}\;\text{equipment}\\ \; & \text{Sum,} & v_{051} \end{array}\right]$$

Similarly, the associated basic-elements *l*_051_ of *l*_05_ can be extension transformed as:

$$T_{051}l_{051}\left\{\begin{array}{l} T_{051}^{\prime }l_{051}=l_{051}^{\prime }=\left[\begin{array}{rrr} \text{Equipment}\;\text{inputs}, & \text{Executing}\;\text{entity}, & \text{Enterprise}\;\text{M}\\ \; & \text{Typology}, & \text{Automated}\;\text{production}\;\text{equipment}\\ \; & \text{Sum}, & v_{051}^{’} \end{array}\right]\\ T_{051}^{\prime \prime }l_{051}=l_{051}^{\prime \prime }=\left[\begin{array}{rrr} \text{Equipment}\;\text{inputs}, & \text{Executing}\;\text{entity}, & \text{Enterprise}\;\text{M}\\ \; & \text{Typology}, & \text{Customised}\;\text{production}\;\text{equipment}\\ \; & \text{Sum}, & v_{051}^{\prime \prime } \end{array}\right]\\ T_{051}^{\prime \prime \prime }l_{051}=l_{051}^{\prime \prime \prime }=\left[\begin{array}{rrr} \text{Equipment}\;\text{inputs}, & \text{Executing}\;\text{entity}, & \text{Enterprise}\;\text{M}\\ \; & \text{Typology}, & \text{Energy-saving}\;\text{production}\;\text{equipment}\\ \; & \text{Sum}, & v_{051}^{\prime \prime \prime } \end{array}\right]\\ T_{051}^{\prime \prime \prime \prime }l_{051}=l_{051}^{\prime \prime \prime \prime }=\left[\begin{array}{rrr} \text{Equipment}\;\text{inputs}, & \text{Executing}\;\text{entity}, & \text{Enterprise}\;\text{M}\\ \; & \text{Typology}, & \text{Intelligent}\;\text{Manufacturing}\;\text{Production}\;\text{Equipment}\\ \; & \text{Sum}, & v_{051}^{\prime \prime \prime \prime } \end{array}\right]\\ \cdots \end{array}\right.$$

By the conductive nature of the transformation, a conductive transformation (*T*_*m*_ ˄ *T*_*n*_ ⋯˄ *T*_*i*_ ⋯) ⇒ *T*_05_ occurs such that $T_{05}l_{051}=l_{05}^{\prime }$:

$$T_{05}l_{05}=\left\{\begin{array}{l} T_{05}l_{051}^{\prime }=l_{05}^{\prime }\\ T_{05}l_{051}^{\prime \prime }=l_{05}^{\prime \prime }\\ T_{05}l_{051}^{\prime \prime \prime }=l_{05}^{\prime \prime \prime }\\ T_{05}l_{051}^{\prime \prime \prime \prime }=l_{05}^{\prime \prime \prime \prime }\\ \cdots \end{array}\right.$$

ln this case, if there exists the dependent function *k*(*v*(*T*_05_*l*_05_)) ≥ 0 of the conditional basic-elements after the extension transformation, then it means that the transformation is feasible.

Divergence analysis of *l*_061_ logistics distribution channels and *l*_062_ logistics personnel obtained from the *l*_06_ logistics assurance correlation analysis of the conditional basic-elements:

$${\text{l}}_{06}∼\left\{\begin{array}{l} {\text{l}}_{061}=\left[\begin{array}{rrr} \text{Distribution}, & \text{Dominating}\;\text{object}, & \text{Logistics}\\ \; & \text{Channel}, & \text{Co-operative}\;\text{Express}\\ \; & \text{Effect}, & {\text{v}}_{061} \end{array}\right]\\ {\text{l}}_{062}=\left[\begin{array}{rrr} \text{Logistics}\;\text{distribution}, & \text{Acting}\;\text{object}, & \text{Co-operative}\;\text{distributor}\\ \; & \text{Distribution}\;\text{effect}, & {\text{v}}_{062} \end{array}\right] \end{array}\right.$$

Similarly, in order to solve the incompatible problem *l*_06_ the related basic-elements *l*_061_ and *l*_062_ can be extension transformed as:

$${\text{T}}_{061}{\text{l}}_{061}\left\{\begin{array}{l} {\text{T}}_{061}^{\prime }{\text{l}}_{061}={\text{l}}_{061}^{\prime }=\left[\begin{array}{rrr} \text{Distribution}, & \text{Dominating}\;\text{object}, & \text{Logistics}\\ \; & \text{Channel}, & \text{Postal}\;\text{Express}\\ \; & \text{Effect}, & {\text{v}}_{061}^{\prime } \end{array}\right]\\ {\text{T}}_{061}^{\prime \prime }{\text{l}}_{061}={\text{l}}_{061}^{\prime \prime }=\left[\begin{array}{rrr} \text{Distribution}, & \text{Dominating}\;\text{object}, & \text{Logistics}\\ \; & \text{Channel,} & \text{Professional}\;\text{logistics}\;\text{and}\;\text{transport}\\ \; & \text{Effect}, & {\text{v}}_{061}^{\prime \prime } \end{array}\right]\\ \cdots \end{array}\right.$$

$${\text{T}}_{062}{\text{l}}_{062}\left\{\begin{array}{l} {\text{T}}_{062}^{\prime }{\text{l}}_{062}={\text{l}}_{062}^{\prime }=\left[\begin{array}{rrr} \text{Logistics}\;\text{distribution}, & \text{Acting}\;\text{object}, & \text{Professional}\;\text{Distributor}\\ \; & \text{Distribution}\;\text{effect}, & {\text{v}}_{062}^{\prime } \end{array}\right]\\ {\text{T}}_{062}^{\prime \prime }{\text{l}}_{062}={\text{l}}_{062}^{\prime \prime }=\left[\begin{array}{rrr} \text{Logistics}\;\text{distribution}, & \text{Acting}\;\text{object}, & \text{Intelligent}\;\text{robot}\\ \; & \text{Distribution}\;\text{effect}, & {\text{v}}_{062}^{\prime } \end{array}\right]\\ \cdots \end{array}\right.$$

By the conductive nature of the transformation, a conductive transformation (*T*_*m*_ ˄ *T*_*n*_ ⋯˄ *T*_*i*_ ⋯) ⇒ *T*_06_ occurs such that $T_{06}l_{061}=l_{06}^{\prime }$:

$$T_{06}l_{06}=\left\{\begin{array}{lll} l_{06}^{\prime }=T_{06}l_{06}^{\prime } & ⇐ & \left\{T_{061}^{\prime }l_{061}\wedge T_{061}^{\prime }l_{061}\right.\\ l_{06}^{\prime \prime }=T_{06}l_{06}^{\prime \prime } & ⇐ & \left\{T_{061}^{\prime }l_{061}\wedge T_{061}^{\prime \prime }l_{061}\right.\\ l_{06}^{\prime \prime \prime }=T_{06}l_{06}^{\prime \prime \prime } & ⇐ & \left\{T_{061}^{\prime \prime }l_{061}\wedge T_{061}^{\prime \prime }l_{061}\right.\\ l_{06}^{\prime \prime \prime \prime }=T_{06}l_{06}^{\prime \prime \prime \prime } & ⇐ & \left\{T_{061}^{\prime \prime }l_{061}\wedge T_{061}^{\prime }l_{061}\right.\\ \cdots & \; & \; \end{array}\right.$$

ln this case, if there exists the dependent function *k*(*v*(*T*_06_*l*_06_)) ≥ 0 of the conditional basic-elements after the extension transformation, then it means that the transformation is feasible.

### 4.6. Superiority evaluation constitutes the optimal resilience enhancement strategy

Through the implementation of the correlation analysis in the extensible analysis of the condition basic-elements of the above Incompatible problem, a large number of tenacity enhancement strategy basic-elements are generated by applying the method in the extension transformation on the relevant condition basic-elements found to have a positive influence, and they are combined to form a more multi-dimensional set of tenacity enhancement extension strategies, and in order to find the optimal set of tenacity enhancement strategies, we calculate the dependent function of the combinations of the tenacity enhancement strategies of supply chain enterprises M. We select the combination with positive and maximum value of the dependent function as the optimal enhancement strategy for supply chain enterprises’ M dependent function, and select the combination with positive and maximum median value of the dependent function *k*(*x*_*i*_) ≥ 0 as the optimal enhancement strategy for resilience enhancement of supply chain enterprise M. There exists a total compatibility function:

$$K\left(p_{0}\right)=\overset{6}{\underset{i=1}{\wedge }}k\left(x_{i}\right)=k_{\text{max}}\left(x_{1}\right)\wedge k_{\text{max}}\left(x_{2}\right)\wedge k_{\text{max}}\left(x_{3}\right)\wedge k_{\text{max}}\left(x_{4}\right)\wedge k_{\text{max}}\left(x_{5}\right)\wedge k_{\text{max}}\left(x_{6}\right)>0$$

By solving the problem of resilience incompatibility of fresh produce supply chain enterprise M and forming a new set of resilience enhancement strategies, and finally combining the calculation and screening of relevant professional knowledge to form a new resilience enhancement strategy for fresh produce supply chain. The resilience enhancement strategy solves the six contradictory problems of insufficient richness of product supply types, lack of product demand, low product supply efficiency, low human resources, weak production and processing equipment, and poor logistics protection ability in six aspects of fresh agricultural products supply chain enterprises.

Enrichment of product supply variety. The increase in variety contributes to the strengthening of supply chain resilience, and the increase in product variety also expands the business and market area, which increases the consumer experience and improves customer satisfaction when meeting different consumer needs. In order to occupy more market share to achieve the goal of profitability, the diversification of product categories in the face of fluctuating market environment, can effectively reduce the risk, to protect the supply chain’s continued stability. Therefore, enterprises can attract funds from more sources to support product research and development activities, and the collection of information on product categories should also obtain data from comprehensive and accurate information platforms to assist in the enrichment of product categories.

Guarantee the supply of products that are in demand. Can guarantee the stability of each link in the supply chain, on-time delivery of the required products to help reduce inventory costs, reduce the excess inventory crisis, and effectively improve the operation of funds, product supply and demand security requirements of high standards of co-operation to promote the development of enterprises to the high resilience of the system to enhance the resilience of the supply of products and reliability, and can be timely to the supply chain to make timely adjustments to the problems that arise in one of the links to reduce the interruption of supply chain and the resulting production It can make timely adjustments to problems in one part of the supply chain and reduce production stagnation and order delays caused by supply chain disruption. Long-term stable product supply also helps to improve the enthusiasm and loyalty of suppliers, and provide better service and support for enterprises. The product supply chain can be transformed from sales strategy and inventory management. Sales strategy, as one of the core elements of product supply, directly affects the operational efficiency and profitability of the enterprise. Adjustment of sales strategy will cause changes in market demand, which in turn affects the ability to supply products. And an effective inventory management system can ensure the stability of product supply, meet customer demand, reduce inventory costs and improve the competitiveness of enterprises.

Improve the efficiency of product supply chain. For the customer side, the improvement of efficiency can make the product faster to the hands of consumers, and enhance consumer satisfaction and trust. On the supply chain side, it also helps to improve competitiveness. Under the environment of global marketisation, efficient product supply can better meet the rapid market demand and flexibly adjust the supply chain links. Therefore, the optimisation of transport and order management processes is an important factor affecting the efficiency of the supply chain. The use of advanced logistics technology to improve the loading rate of transport means can effectively shorten the transport time and increase the number of transports, which can well enhance the transport efficiency. Secondly, the perfect optimisation of the order guarantee can understand the change of customer’s demand in time, and improve the accuracy of the order and the timeliness of the product delivery.

Optimise personnel training mode. Through the scientific, reasonable and systematic training of managers and employees in supply chain enterprises, employees can not only master more professional knowledge and skills, but also enhance teamwork ability and improve work enthusiasm, thus promoting the overall optimisation and upgrading of supply chain resilience. Therefore, personnel training is an important means to enhance the supply chain resilience, but also a key factor in the sustainable development of enterprises.

Optimise production and processing equipment. Improvement of production and processing equipment can reduce production costs, improve the efficiency and flexibility of the production line, reduce carbon emissions in the production and processing process and pollution of the environment, but also to ensure the safety of the production and processing process, to reduce the cost of enterprise consumption and to ensure the stability of its operations and competitiveness, but also to protect the safety and health of employees, to reduce the production of hidden safety hazards and the risk of delays caused by insufficient productivity, and to greatly enhance the resilience of the supply chain. Greatly enhance the resilience of the supply chain.

Enhance the ability of logistics security. The choice of logistics distribution channels affects the transport efficiency and transport costs, the attributes of different products should choose different and effective channels, the appropriate distribution channels can minimise the waste of time and cost, improve the efficiency of logistics distribution, secondly, the level of professional skills of the distribution staff also affects the quality of logistics distribution, professional and efficient distribution staff will pay attention to the nature of the distribution products and product protection, and have a better communication skills and service consciousness. Better communication skills and service consciousness, can communicate effectively with customers, timely solution to deal with unexpected problems. Therefore, enterprises in the logistics distribution should pay attention to channel selection and personnel training, and constantly improve their own logistics management capabilities.

## 5. Conclusions

Nowadays, facing the problem of unstable factors such as international society and ecological and climatic environments, fresh agricultural products are essential food sources in people’s daily life and account for an important part of the national economy, market consumers’ awareness of the stability and sustainability of the quality of the supply of fresh agricultural products has been gradually strengthened, and the national policy has also given important instructions on the various aspects of the supply chain of fresh agricultural products, therefore, the resilience of the fresh agricultural products supply chain has become the main goal of supply chain development. Therefore, the resilience of fresh produce supply chain has become the main goal of supply chain development. However, when improving the resilience, we should consider the resources, structure, environment and other complex elements of the supply chain, evaluate the resilience state of the supply chain from the scientific, efficient, multi-dimensional and quantitative perspectives, and implement optimisation strategies for the resilience problems. In this paper, with the help of the theoretical method of topology, through the data calculation and analysis of the evaluation of supply chain resilience, the index of low resilience is expanded and analysed with extension transformations, and the method of generating extension strategies for the conditioned basic-elements of the nuclear problem is constructed.

### 5.1. Impact

According to the characteristics and importance of the fresh produce supply chain, this paper selects the three facets of fresh produce resilience, capital resilience and internal resilience to construct the enterprise resilience evaluation system, and through the calculation of the index weights and correlation function values, it applies quantitative analysis to objectively and efficiently evaluate the resilience state of the fresh produce supply chain enterprises, and explores the incompatibility of the enterprise’s resilience enhancement goal with the contradiction of the existing conditions.

In order to reach a topical solution to the incompatible problem of improving the resilience of fresh produce supply chain, this paper expands the analyses and topical transformations of the existing condition basic-elements, which gives more inspiration to the enterprise M to solve the incompatible problem of the current resilience condition basic-elements.

The practical significance of this study is to give fresh produce supply chain enterprises a more multi-dimensional perspective to analyse and solve the deficiencies in the supply chain operation process, to better cope with the complexity and uncertainty brought by the supply chain, and to guarantee the national strategic deployment and stable social development with efficient and transparent supply chain resilience.

### 5.2. Limitations and future prospects

Although this paper generates resilience enhancement strategies for fresh produce supply chain enterprises from a multidimensional and scientific perspective, there are still some shortcomings, this study is subject to subjective conditions, it is difficult to construct a resilience indicator system with a comprehensive, scientific and objective system in terms of indicator selection, and in the generation of resilience enhancement strategies, the basic-elements are subjective and easy to formulate unclear, expanding the analysis of the condition of basic-elements and expanding the transformations. The generation efficiency is not high, and many conditional basic-elements that cannot solve the compatibility problem will be generated.

In summary, in the future, in the construction of supply chain resilience index system of fresh agricultural products, the artificial intelligence recommendation system can be used to screen and identify the indexes with the advantages of objectivity and high efficiency, and optimize the process of expanding analysis and extension transformation, so that the intelligent system can screen the more relevant and influential basic-elements, and improve the effectiveness and scientificity of the resilience strategy generation.

## Supporting information

S1 Data (XLSX)
